# Individual differences in perceptual abilities in medical imaging: the Vanderbilt Chest Radiograph Test

**DOI:** 10.1186/s41235-017-0073-4

**Published:** 2017-09-20

**Authors:** Mackenzie A. Sunday, Edwin Donnelly, Isabel Gauthier

**Affiliations:** 1 Department of Psychology, Vanderbilt University, 226 Wilson Hall, Nashville, TN 37204 USA; 2 Department of Radiology and Radiological Sciences, Vanderbilt, USA

**Keywords:** Vision, Perception, Detection, Diagnostic

## Abstract

Radiologists make many important decisions when detecting nodules on chest radiographs. While training can result in high levels of performance of this task, there could be individual differences in relevant perceptual abilities that are present pre-training. A pre-requisite to address this question is a valid and reliable measure of such abilities. The present work introduces a new measure, the Vanderbilt Chest Radiograph Test (VCRT), which aims to quantify individual differences in perceptual abilities for radiograph-related decision-making in novices. We validate the relevance of the test to diagnostic imaging by verifying radiologists’ superior performance on the test compared to novices’. The final VCRT version produces scores with acceptable internal consistency. Then, we investigate how the VCRT can be used in future research by evaluating how the test relates to extant measures of face and object recognition ability. We find that the VCRT shares a small but significant portion of its variance with a measure of novel object recognition, suggesting that some aspect of VCRT performance is driven by a domain-general visual ability.

## Significance statement

This work presents a new measure of lung-nodule detection ability for use in research investigating radiological expertise and training. Additionally, the work presents evidence that there may be a general visual ability relevant to detecting nodules in thoracic radiographs.

## Background

In the United States, becoming a thoracic radiologist usually requires 4 years of medical school, 1 year of internship, 4 years of residency and one additional year of a thoracic radiology fellowship. This training qualifies radiologists to make expert decisions of vital importance in medical treatment, but studies have documented a non-negligible level of errors in these decisions (Goddard, Leslie, Jones, Wakeley, & Kabala, 2001; Manning, Ethell, & Donovan, 2004). A better understanding of the various influences on these decisions could help to lessen this error rate. The bulk of radiological expertise research has focused on the relation between search patterns and nodule detection (specifically, to address whether radiologists engage in holistic processing (Donovan & Litchfield, 2013; Drew, Evans, Võ, Jacobson, & Wolfe, 2013; Kundel, Nodine, Conant, & Weinstein, 2007). Most of this research has investigated visual search patterns of radiologists to show that radiologists scan radiological images differently from novices (Bertram, Helle, Kaakinen, & Svedström, 2013; Kundel, Nodine, & Carmody, 1978; Mello-Thoms et al., 2005). However, other work has shown that experts can rapidly identify nodules at above chance rates in short durations that would only allow a few eye movements (durations as short as 200 ms in Kundel & Nodine, 1975; for other work see Oestmann et al., 1988; Mugglestone, Gale, Cowley, & Wilson, 1995; Kundel et al., 2007; Carmody, Nodine, & Kundel, 1981), suggesting that expertise may partly rely on aspects of perceptual processing that do not require visual search.

Another question addressed in radiological research has been whether radiological expertise generalizes to other tasks and domains (Beck, Martin, Smitherman, & Gaschen, 2013; Nodine & Krupinski, 1998; Sowden, Davies, & Roling, 2000). The results of this work have been inconclusive so far, with some work showing that lower-level perceptual abilities, such as contrast sensitivity, are enhanced in radiologists (Sowden et al., 2000), but more complex skills like visual search (Nodine & Krupinski, 1998) and visual working memory (Beck et al., 2013) are unaffected by acquiring radiological expertise.

Within all of this work, individual differences in performance are sometimes noted (Donovan & Litchfield, 2013) but rarely discussed. Variability in radiologists’ performances may occur for several reasons, including differences in decision-making (Donovan & Litchfield, 2013) and perceptual abilities (Bass & Chiles, 1990). In turn, these abilities may be influenced by variability in training and experience or pre-existing individual differences in perceptual abilities. These influences of individual differences have remained unexplored, and even when radiological performance is explicitly measured, most studies do not focus on the psychometric properties of the task, including its reliability (Bass & Chiles, 1990; Harley et al., 2009). The general goal of our study is to develop a test capable of measuring such pre-existing individual differences to then determine how these individual differences might relate to object recognition abilities.

Because the study of individual differences in high-level vision is a recent development, it is unsurprising that pre-existing individual variability in the field of radiology has not been considered. People likely underestimate the extent to which individuals in the normal population vary in perceptual ability, but recent studies have shown large individual differences in perceptual processing of faces, of various familiar object categories, and even of novel objects (Dennett et al., 2012; Duchaine & Nakayama, 2006; McGugin, Richler, Herzmann, Speegle, & Gauthier, 2012; Richler, Wilmer, & Gauthier, 2017). Given the recentness of these findings, individual differences in novice radiological detection abilities have been overlooked.

Our goal in creating a measure of perceptual abilities relevant to the domain of chest radiographs is to examine whether variability in novice perceptual abilities determines how much an individual can benefit from experience, and, ultimately, how they will differ as experts. Critically, we design our test for use with a novice population. To that end, we use a four-alternative, forced-choice method with a single nodule present on each target to tap into perceptual processing, in contrast to more complicated tasks in which subjects do not know how many nodules may be present, involving more complicated decision-making processes (Bass & Chiles, 1990; Donovan & Litchfield, 2013). A great deal of research in radiology comes from the tradition of visual search (Bertram et al., 2013; Carmody et al., 1981; Drew et al., 2013; Kundel et al., 1978). In most classic visual search studies, the target is well-specified and the difficulty comes from localizing it among distractors that possess similar features. The present work was inspired by a different tradition, studies in category learning and object recognition (Palmeri & Gauthier, 2004), in which categories are more probabilistically defined, and in some cases, have to be learned by subjects through trial and error. Therefore, here we are less interested in the ability to localize nodules following instruction on what they look like, and more interested in subjects’ abilities to learn the features of suspicious nodules from examples. Ultimately, the processes involved in category learning and in visual search are both likely to be relevant to real-world radiological training.

In addition, because most people have little to no familiarity with nodule detection in chest radiographs (compared to recognition of faces, cars, planes, etc.), we measure the extent to which nodule detection is predicted by performance in novel object recognition. A recent test of novel object recognition memory (Novel Object Memory Test (NOMT); Richler et al., 2017) measured an ability distinct from general intelligence which generalized across visually different novel objects categories (*r* ^2^ = .23) and was distinct from face or car recognition abilities (*r* ^2^ = .10, Richler et al., 2017). Given this, we correlate our chest radiograph test with tests of novel object recognition and of face recognition ability (the Cambridge Face Memory Test (CFMT), Duchaine & Nakayama, 2006) to determine if our measure of nodule detection ability shares more variance with a novel object measure than a face recognition measure (as we predict). To be clear, our goal is not to determine whether expert radiological detection is the same as expert object recognition. Rather, our main aim in testing these relations is to demonstrate how our new test might be used in future research to determine if a domain-general object recognition ability is relevant to radiological expertise. If the new measure we create is very highly correlated with performance on the NOMT (which is possible since chest radiographs are to some extent novel objects to novices), this would suggest a domain-specific test like the one that we have created is not necessary to measure pre-existing perceptual abilities relevant to these decisions.

In three studies, we present our new nodule detection test and then begin to explore important properties of the test. We honed our new test to produce acceptable reliability in study 1, and then assessed the test’s validity by measuring how well medical professionals performed on the test (study 2). In study 3, we asked if there was any shared variance between our nodule detection test and a face and object recognition measure, to see if our test might be useful in determining how a domain-general ability may contribute to real-world skills like nodule detection.

## Study 1

To create a measure of lung-nodule detection ability, we developed the Vanderbilt Chest Radiograph Test (VCRT). Because low reliability can attenuate the observed correlation between two measures (Nunnally, 1970), it is crucial that we develop a test that produces reliable scores (keeping in mind that reliability is not a test property and must be evaluated with each new dataset). Through several iterations we honed the test to produce reliable scores with a novice population.

### Methods

#### Subjects

For the first version of the VCRT, 50 subjects were recruited online from Amazon Mechanical Turk and compensated US$0.50. For all experiments, only subjects with US IP addresses and at least 95% of their previous Amazon Mechanical Turk tasks accepted were eligible to participate. Subjects were asked to rate their expertise with “chest X-rays” on a scale from 1 to 9. Two subjects were excluded for failure to follow instructions, leaving 48 subjects for analysis (18 male, mean age = 35.33 years). For the second VCRT version, 49 subjects were recruited and compensated US$0.50. One subject was excluded for incorrectly answering both catch trials, and of the 48 remaining subjects, 16 were male (mean age = 38.65 years). One hundred and nineteen subjects were recruited to complete the final VCRT followed by an additional test discussed in study 3 (Novel Object Memory Test) and were compensated US$0.75 for completing both. Ten of these subjects were excluded for failure to follow instructions, leaving 108 subjects (39 male, mean age = 38.51 years). This study and all following studies were conducted under approval by the Vanderbilt University Institutional Review Board and informed consent was obtained for each subject.

#### Stimuli

Stimuli were chest radiographs of 212 individuals (with any identifying information removed). Of these, 106 chest radiographs contained cancerous nodules (no image contained multiple nodules) and 106 were nodule free. All nodules were confirmed in a follow-up computed tomography (CT) scan to be non-calcified nodules and the nodules had a mean diameter of 25.3 mm (standard deviation (SD) = 12.6 mm, range = 7.0–67.5 mm). Nodules were identified by one of the authors, a thoracic radiologist with over 20 years of experience reading chest radiographs. Images were cropped to a 1.3:1 ratio and converted to grayscale. Other than this, images were not altered, so any inorganic elements (pacemakers, surgical screws, shadows from bed gurneys, etc.) were included. In this way, we hoped to keep the chest radiograph images as similar as possible to images seen by radiologists in the field, thereby maximizing the test’s construct validity. Nodules appeared 49 times in the left lung and 31 times in the right lung. Though this may have produced a slight left-bias, because each individual sees the same stimuli, this left-bias would not confound the measured individual differences.

#### Procedure

**Fig. 1:**
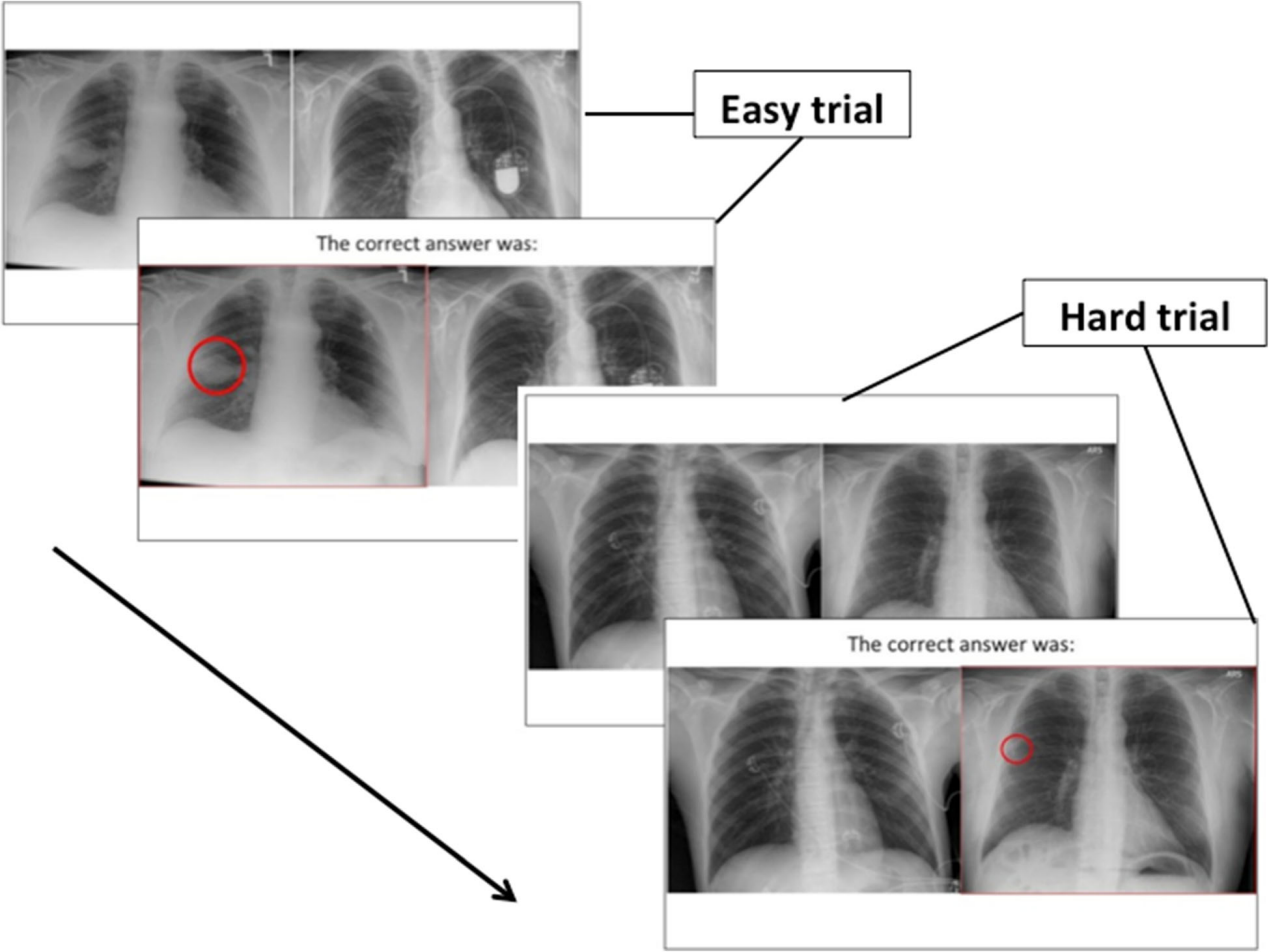
Example trials from the Vanderbilt Chest Radiograph Test (VCRT). Subjects responded by clicking on the nodule and were then given feedback and shown the nodule for 2000 ms. The upper trial is an example of an easy trial and bottom trial is an example of a more difficult trial

The initial test began with instructions and two practice trials, followed by 106 total trials (two of which were catch trials). Each practice trial was identical to the experimental trials except the feedback was accompanied with text saying “here is the nodule” so that the subjects understood the feedback. Other than these two practice trials, subjects were given no specific instructions about the nodules but were told that they could learn from the feedback. On each trial, subjects viewed two chest radiographs, presented horizontally (Fig. 1). Subjects were instructed to “guess which of the four lungs has a cancerous nodule” and to indicate their response by clicking on the location where they believed the nodule was. Responses were un-speeded. Subjects were scored as correct if they clicked on the correct quarter of the screen (as divided horizontally into four vertical sections, corresponding to each of the four lungs). We did not record the exact location of the click but only the selection of the chosen lung. Because of this, and the fact that we did not purposefully manipulate any of our stimuli properties (number of targets, contrast, etc.), we did not design our task to be a standard search task, though subjects were asked to search through images for a nodule. A chance level of 25% was considered sufficient to measure individual differences among untrained novices. Following each response, subjects received feedback for 2000 ms, during which the correct radiograph image (right or left) was *outlined in red* and the nodule *circled in red* (Fig. 1). Our decision to include feedback on every trial was in part based on pilot data showing that when no feedback was given to subjects, performance was at chance.^1^ We did not intend for the test to be a training task, but instead wanted to measure how well novice subjects can learn from exposure and feedback to detect lung nodules. We did not design the task to be used for training or to measure the efficacy of a training protocol, in fact the task may be too easy for experts and may be more useful in assessing whether individual differences before training predict how well individuals benefit from training. On the two catch trials, one chest radiograph was presented along with a landscape scene, and any response choosing the chest radiograph (either lung) was coded as correct. The procedure used in the second VCRT version and the final VCRT were the same as the initial version. Trials were always presented in the same order to reduce contribution of order variance in the measurement of individual differences.

A limitation of testing people online is that some of the variability in performance could be attributed to different testing conditions (not only screen differences, but also ambient light and other factors such as noise in the room, presence of other people, etc.). This is a tradeoff against the lack of subject variability that arises when only undergraduate students are tested in the laboratory (in this case, there may be less variability due to testing conditions, but there may also be less person variability). To reduce variability due to testing conditions, we instructed subjects not to complete the test on a handheld screen, and, in the final VCRT version, had subjects perform a contrast check before the test. This contrast check consisted of three trials preceding the practice trials that required subjects to choose a low-contrast diagonal Gabor patch from a set of three patches (the other two being solid). This test was meant to ensure that subjects were completing the test on a screen with sufficient contrast. If subjects did not correctly answer this contrast check, they were instructed to increase their screen contrast. Prior research with the CFMT reveals that tests can perform similarly online and in the laboratory (even at the level of individual trial information, Cho et al., 2015) and that person characteristics (such as gender) that predict differential performance on some tests do so in both online and laboratory samples (Ryan & Gauthier, 2016), demonstrating that testing condition variance does not overshadow true individual differences. Nonetheless, future work should validate the VCRT under more controlled conditions.

### Results

The initial VCRT version had an average accuracy of 45.00% (SD = 8.15%) and produced an internal consistency of *α* = .736. We examined items having a low correlation between item responses and subjects’ total scores (which were thus relatively uninformative). For 44 items, we replaced the distractor images (chest radiographs with no nodules), with a different distractor image. This second version of the test had an average accuracy of 48.70% with less variance than the first version (SD = 6.65%). In addition, internal consistency was also lower than the initial version (*α* = .609).

We further sought to improve reliability by taking the 78 trials from the second version that produced the highest correlation between item responses and subjects’ total scores (i.e., the most informative trials), while attempting to maximize the range of difficulty in test items, to create the final VCRT. For this final VCRT, we also ordered trials from easiest to most difficult based on the item accuracies produced in the second VCRT version. The final VCRT version has 80 trials total (including two catch trials), and takes approximately 20 min to complete. This final version had average accuracy of 53.00% (SD = 10.13%) and we observed acceptable reliability in our sample (*α* = .799). This final version is available online at http://gauthier.psy.vanderbilt.edu/resources/and the data are available at https://figshare.com/articles/Data_for_Sunday_et_al_2017/5278234.

### Discussion

We developed the VCRT to measure the ability of novices to learn to identify lungs that contain suspicious nodules in chest radiographs, based on feedback. Based on the first two versions of the VCRT, we created the final VCRT, which produces reliable scores of chest nodule detection ability. Though our test has good face validity, it is important to critically evaluate the construct validity to ensure that our test is measuring its targeted construct, which we do in study 2.

## Study 2

To validate our new test of nodule detection in chest radiographs, we asked radiologists and radiological students to complete the test. If the VCRT taps into a construct used by radiologists to make actual determinations about the presence of cancerous nodules, then we would expect medical professionals to perform well on the test.

### Methods

#### Subjects

We recruited five medical professionals to complete the final VCRT version (hereafter referred to as the VCRT). Subjects who completed the task were given a 1-in-5 chance to win US$20.00. Two subjects had completed thoracic radiology fellowships and the remaining three were radiology residents (three male, mean age = 37.4 years).

#### Procedure

The final version of the VCRT, with 80 trials total, was used.

### Results

Average VCRT accuracy of the expert group was 81.54% (SD = 6.32%), which was significantly greater than the non-medical professionals’ performance reported in study 1 (*t*(3) = 9.0; *p* = .002, *d* = 3.38).

### Discussion

Our medical professionals performed well on the VCRT. We believe that these medical professionals performed well mostly because of their extensive training and experience with reading chest radiographs. However, it is certainly possible that their performance could be due to increased motivation, a difference in strategy, or something else. A number of other differences could have contributed to the above average performance of the medical professionals, so further investigation is needed to elucidate possible causes for their superior performance.

Regarding our goal of assessing the validity of our test, we can definitively say that it would have been concerning if these medical professionals performed poorly, or even in the same range as novices on our test. However, this sample of medical professionals was superior in their ability to detect nodules compared to novice observers. This supports the VCRT as a valid measure of nodule detection ability in chest radiographs, although it is impossible to know whether they achieved superior performance using a qualitatively different strategy from novices. For now, these results serve to better characterize the VCRT and open the door for further research aimed at validating this measure.

## Study 3

With the aim of creating a measure of nodule detection in chest radiographs, we developed the VCRT. Our purpose in creating such a measure is to provide a useful tool for future work studying perceptual individual differences as may be relevant to medical training. However, one possibility is that because chest radiographs are essentially novel objects to novices (as compared with domains like cars and faces) our test will essentially tap into the same ability as existing tests that measure perceptual abilities with novel objects. Therefore, we decided to quantify the overlap between the ability measured by the VCRT and an existing test of recognition for novel objects. Our purpose here is not to draw conclusions about the nature of these domains based on the specific mechanisms involved in each of these tasks but rather to better understand how the ability to learn how to identify suspicious chest nodules based on feedback relates to face and novel object recognition ability. While the VCRT involves a purely perceptual task, the CFMT and NOMT tasks include both a perceptual component (to encode the stimuli) and a memory component. Previous work provided evidence that the NOMT measures a domain-general visual ability that is independent from general intelligence and memory span (Richler et al., 2017). The CFMT and NOMT are existing measures of high-level visual abilities that have been found to correlate with performance on other perceptual tasks in past research – importantly, any correlation between these each of these tasks and the VCRT cannot be attributed to similarity of task format and would, therefore, be more likely due to task-general visual ability.

This relation is interesting in light of recent evidence for domain-general visual abilities relevant to object recognition, as expressed by common variance between tests of familiar and novel object recognition (Richler et al.: Individual Differences in Object Recognition, submitted; Van Gulick, McGugin, & Gauthier, 2016). Additionally, novel object recognition shows some limited shared variance (*r* ^2^ = .10; Richler et al., 2017) with face recognition, as measured by the CFMT (Duchaine & Nakayama, 2006). Because chest radiographs are likely closer to novel than familiar objects within a novice population, we expected VCRT performance to show a stronger correlation with a novel object recognition measure than with a face recognition measure. Finding this would indicate that some of the VCRT performance relies on the same ability relevant to discriminating novel objects across different viewpoints, providing further evidence for a domain-general visual ability. We also expected to replicate the small but significant correlation between the CFMT and NOMT.

### Methods

#### Subjects

One hundred and nineteen subjects were recruited to complete the VCRT followed by the NOMT and compensated US$0.75 (as described in study 1). The 108 subjects (38 male, mean age = 38.41 years) who were not excluded from analyses in study 1 were given the opportunity to complete the CFMT for an additional US$1.00. Of the 75 subjects who chose to complete the CFMT, 23 were male (mean age = 39.24 years). Additionally, the five medical professionals from study 2 were given the opportunity to complete the NOMT and four did so and were thus compensated US$10.00.

#### VCRT

The final version of the VCRT (also used study 2) was used.

#### Cambridge Face Memory Test (CFMT)

In the CFMT (Duchaine & Nakayama, 2006), subjects studied six Caucasian grayscale male target faces and then had to correctly identify the target face presented with two foil faces on each trial. The first block showed target faces in the studied viewpoint (18 trials), and in the second block subjects identified the target across variations in lighting and viewpoint (30 trials). For the third block (24 trials), Gaussian noise was added to novel target images. Here, we used the long version of the CFMT (Russell, Duchaine, & Nakayama, 2009), so there was an additional final block (30 trials), which was designed to be the most difficult, with uncropped faces in profile and additional noise added. Subjects studied the target images between each block and responses were un-speeded.

#### Novel Object Memory Test (NOMT)

The NOMT is a test of object recognition ability that minimizes the influence of experience by using computer-generated novel objects with which subjects have no experience. The test has produced reliable scores in a normal population tested online (Richler et al., 2017) and shows convergent validity due to its correlation with similar tasks with other novel categories (*r* ^2^ = .23). The test follows a procedure modeled after the CFMT, where six novel objects are learned and then tested with a three-alternative forced choice in subsequent trials. In the NOMT, there are 54 trials following the learning phase (in which feedback is given), in which objects have to be recognized across small variations in viewpoint. Here, we use the novel object category called Ziggerins (Wong, Palmeri, & Gauthier, 2009, Fig. 2).

**Fig. 2:**
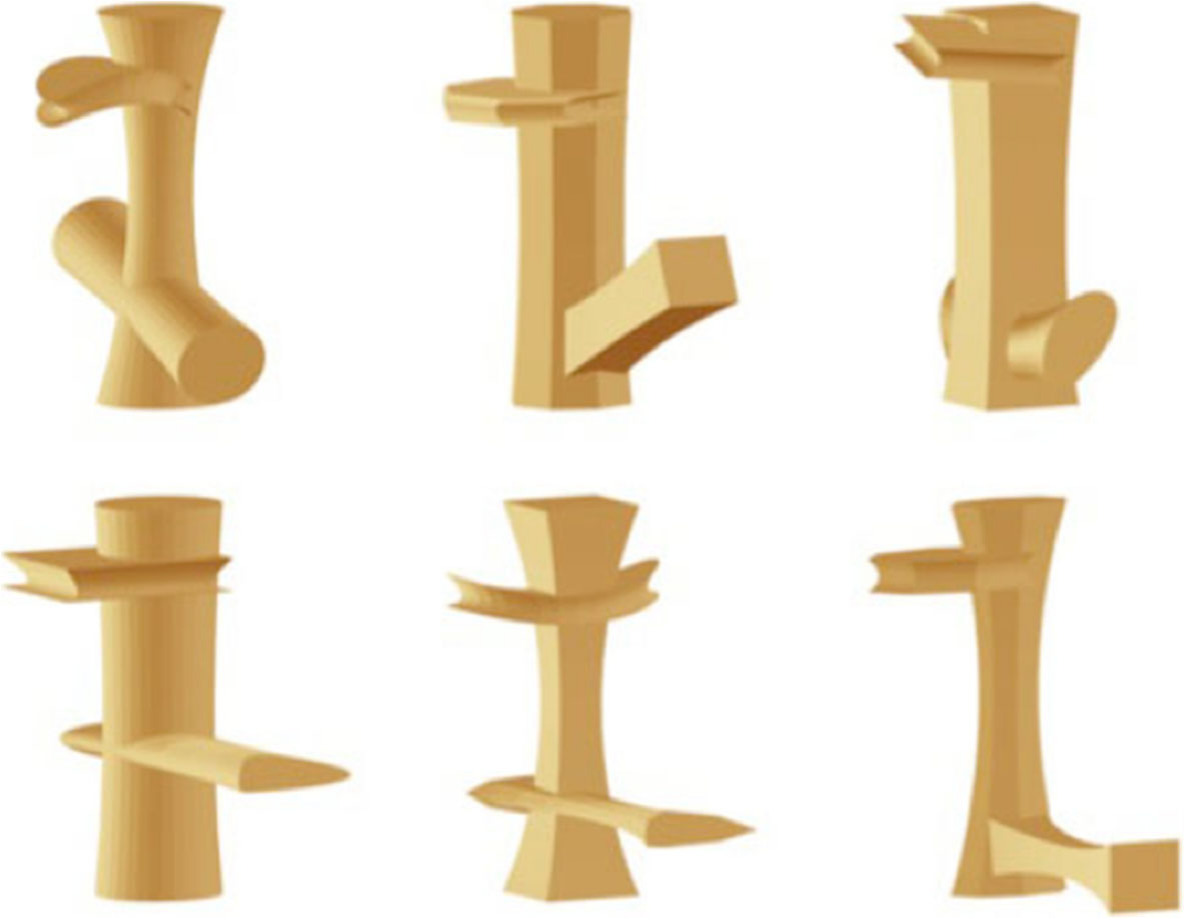
Examples of six Ziggerin stimuli used on the Novel Object Memory Test

### Results

**Fig. 3:**
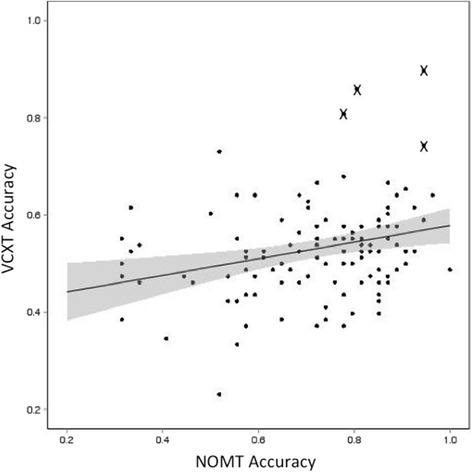
Scatterplot of Novel Object Memory Test (NOMT) and Vanderbilt Chest Radiograph Test (VCRT) accuracies (*N* = 112, medical professionals’ data points marked with X’s). Shaded region indicates 95% confidence intervals

With all 112 subjects (108 from study 1 plus the four medical professionals) who completed the VCRT and NOMT, average NOMT accuracy was 71.54% (SD = 16.73%). Average total time to complete the VCRT (including instructions and practice trials) for online subjects was 26.97 min (SD = 8.9 min) and the average response time on a single trial was 6.93 s (SD = 3.06 s). The average response time of the four medical professionals was 6.77 s (SD = 5.79 s), which did not differ significantly from the online subjects (*t*(110) = 0.01, *p* = .99). Self-reported chest radiograph expertise (on a scale from 1 to 9, M = 3.46, SD = 1.69) from the online subjects did not correlate with VCRT accuracy (*r*_108_ = .07, 95% CI [−.12, .25], *r* ^2^ = .004, *p* = .48), so all online subjects were included. Both tests produced acceptable reliabilities (VCRT *α* = .799; NOMT *α* = .960). There was a significant correlation between performance on the VCRT and NOMT (*r*_108_ = .23, 95% CI [.04, .40], *r* ^2^ = .05, *p* = .02, Fig. 3). This correlation increased somewhat with the four medical professionals included (*r*_112_ = .28, 95% CI [.10, .44], *r* ^2^ = .08, *p* = .003, Fig. 3).

Average CFMT accuracy was 52.00% (SD = 12.25%) and the CFMT also showed good internal consistency (*α* = .839). As in prior work, the CFMT and NOMT were significantly correlated (*r*_75_ = .29, 95% CI [.07, .48], *r* ^2^ = .08, *p* = .01). However, the CFMT and VCRT did not correlate significantly (*r*_75_ = .12, 95% CI [−.11, .34], *r* ^2^ = .01, *p* = .3), and moreover, the VCRT was not significantly more correlated with the NOMT than with the CFMT (Steiger *Z* = .80. *p* = .42).

### Discussion

With the creation of a reliable measure of lung-nodule detection ability in novices, we investigated how this ability relates to other high-level visual abilities measured in recent work. We find that the VCRT shares a small but significant amount of variance with a measure of novel object recognition ability, although we did not have sufficient power to demonstrate that there was more variance than the test shared with face recognition ability. Future efforts should include additional domains and other task formats to better characterize the relation between the ability measured in the VCRT and object recognition abilities. Importantly, given its reliability coupled with the present results, the VCRT appears to measure variation between individuals that is distinct from what is measured in these existing tasks.

Interestingly, and despite the modest correlation between the VCRT and the NOMT, the four medical professionals also performed well on the NOMT. The two radiological residents scored above average (80.56% and 77.78%) and the two subjects who had completed thoracic radiology fellowships scored over 1 SD above average (both 94.44%). Given the small sample size of medical professionals we have, this is merely an intriguing observation. It could be attributed to superior motivation in our experts, but it is also possible that only individuals with very good domain-general visual skills choose and succeed in medical imaging. More work with larger samples and additional tasks is needed to better understand novel object recognition abilities in expert radiologists, but our work suggests the utility of using tests of object recognition ability in expert radiologists, in addition to the visual search and working memory tasks that have been used in prior research (Donovan & Litchfield, 2013; Nodine & Krupinski, 1998; Beck et al., 2013). Generally, this work provides a starting point for further research investigating how the VCRT relates to other measures.

## Conclusions

In three studies, we present a new measure of lung-nodule detection ability (the VCRT), validate this measure and then assess how the measure relates to object recognition abilities. Our test provided reliable measurements of novices’ detection of cancerous lung nodules within chest radiographs. We also found that radiologists performed above average on our test (average z-score = .92), providing some evidence that the test taps into an ability that is high in expert radiologists.

Our long-term goal is to determine whether this test could predict outcomes of diagnostic radiological training. With this goal in mind, we find that our test shares a small amount of variance with a novel object recognition measure, tentatively suggesting that a small but significant amount of variation in VCRT performance may be accounted for by a domain-general recognition ability. Though we might attribute this shared variance to the fact that chest radiographs can be considered novel to the novice subjects (though how novel chest radiographs are to subjects is an unexplored question), we also find that the small sample of experts show above-average NOMT performance. Thus, we are hesitant to conclude that the variance shared between the VCRT and NOMT is entirely driven by the novelty of each domain (since chest radiographs are not novel to radiologists). Instead, we cautiously conclude that some aspect of the ability measured by the NOMT is also relevant to the ability to detect nodules in chest radiographs. Critically, these results highlight the importance of using multiple visual tests when comparing experts and novices. For instance, one study found that experts outperformed novices on a transfer task meant to tap into similar processes as radiograph readings (Sowden et al., 2000), but did not include a control task (like the NOMT) to measure more distant visual processing. Given the result in study 3, it is difficult to determine whether the experts in that study outperformed novices in the transfer task because of their radiological expertise (as was concluded in the study), because of a domain-general advantage, or a combination of the two. Thus, in addition to providing a new test that can be used to measure chest radiograph nodule detection in novices, this work also suggests that studies comparing novices and experts in domain-specific tasks will benefit from the inclusion of visual tests that tap into a varied set of visual abilities (ideally, some visual abilities in which differences are predicted and some in which no differences are predicted).

We already know that experts can demonstrate superior perceptual performance (Russell et al., 2009; Curby, Glazek, & Gauthier, 2009) and considerable work in perceptual learning demonstrates that such abilities can be acquired through practice (Gauthier, Williams, Tarr, & Tanaka, 1998; Jiang et al., 2007; Op de Beeck, Baker, DiCarlo, & Kanwisher, 2006; Tanaka, Curran, & Sheinberg, 2005; Rossion, Gauthier, Goffaux, Tarr, & Crommelinck, 2002; Wong et al., 2009; Sagi, 2011). A new research program rooted in individual differences could help us to understand whether some individuals can learn faster than others, and whether pre-training abilities like that measured by the VCRT places a limit on one’s ultimate level of performance.

## Abbreviations

CFMT: Cambridge Face Memory Test
NOMT: Novel Object Memory Test
VCRT: Vanderbilt Chest Radiograph Test

## Footnotes

1 For the no-feedback 3AFC pilot, the average accuracy of 100 subjects was 36.96% (SD = 5.34%).
